# GenClust: A genetic algorithm for clustering gene expression data

**DOI:** 10.1186/1471-2105-6-289

**Published:** 2005-12-07

**Authors:** Vito Di Gesú, Raffaele Giancarlo, Giosué Lo Bosco, Alessandra Raimondi, Davide Scaturro

**Affiliations:** 1 Dipartimento di Matematica ed Applicazioni, Universitá di Palermo, Via Archirafi 34, 90123 Palermo, Italy

## Abstract

**Background:**

Clustering is a key step in the analysis of gene expression data, and in fact, many classical clustering algorithms are used, or more innovative ones have been designed and validated for the task. Despite the widespread use of artificial intelligence techniques in bioinformatics and, more generally, data analysis, there are very few clustering algorithms based on the genetic paradigm, yet that paradigm has great potential in finding good heuristic solutions to a difficult optimization problem such as clustering.

**Results:**

*GenClust *is a new genetic algorithm for clustering gene expression data. It has two key features: (a) a novel coding of the search space that is simple, compact and easy to update; (b) it can be used naturally in conjunction with data driven internal validation methods. We have experimented with the FOM methodology, specifically conceived for validating clusters of gene expression data. The validity of *GenClust *has been assessed experimentally on real data sets, both with the use of validation measures and in comparison with other algorithms, i.e., *Average Link, Cast, Click *and *K-means*.

**Conclusion:**

Experiments show that none of the algorithms we have used is markedly superior to the others across data sets and validation measures; i.e., in many cases the observed differences between the worst and best performing algorithm may be statistically insignificant and they could be considered equivalent. However, there are cases in which an algorithm may be better than others and therefore worthwhile. In particular, experiments for *GenClust *show that, although simple in its data representation, it converges very rapidly to a local optimum and that its ability to identify meaningful clusters is comparable, and sometimes superior, to that of more sophisticated algorithms. In addition, it is well suited for use in conjunction with data driven internal validation measures and, in particular, the FOM methodology.

## Background

In recent years, the advent of high density arrays of oligonucleotides and cDNAs has had a deep impact on biological and medical research. Indeed, the new technology enables the acquisition of data that is proving to be fundamental in many areas of the biological sciences, ranging from the understanding of complex biological systems to clinical diagnosis (see for instance the Stanford Microarray Database [1]).

Due to the large number of genes involved in each experiment, cluster analysis is a very useful exploratory technique aiming at identifying genes that exhibit similar expression patterns. This may highlight groups of functionally related genes. This leads, in turn, into two well established and rich research areas. One deals with the design of new clustering algorithms and the other with the design of new validation techniques that should assess the biological relevance of the clustering solutions found. Despite the vast amount of knowledge available in those two areas [2-7], gene expression data provide unique challenges, in particular with respect to internal validation criteria. Indeed, they must predict how many clusters are really present in a data set, an already difficult task, made even worse by the fact that the estimation must be sensible enough to capture the inherent biological structure of functionally related genes. As a consequence, a new and very active area of research for cluster analysis has flourished [8-12]. Techniques in artificial intelligence find wide application in bioinformatics and, more in general, data analysis [13]. Although clustering plays a central role in these areas, very few clustering algorithms based on the genetic paradigm are available [14,15], yet such a powerful paradigm [16] has great potential in tackling a difficult optimization problem such as clustering, in particular for high dimensional gene expression data.

Here we give a genetic algorithm, referred to as *GenClust*, for clustering gene expression data and show experimentally that it is competitive with either classical algorithms, such as *K-means *[5], or more innovative and state-of-the-art ones, such as *Click *[17] and *Cast *[18]. Moreover, the algorithm is well suited for use in conjunction with data driven internal validation methodologies [8,9,11,12] and in particular FOM, which has received great attention in the specialized literature [19]. Finally, we mention that *GenClust *is a generic clustering algorithm that can be used also in other data analysis tasks; e.g., sample classification, exactly as all other algorithms we have used here for our study.

## Implementation

### Clustering as an optimization problem

Let *X *= {*x*_1_, *x*_2 _..., *x*_*n*_} be a set of elements, where each element is a *d*-dimensional vector. In our case, each gene is an element *x *∈ *X*, and *x*_*i *_is the value of its expression level under experimental condition *i*. Given a subset *Y *= {*y*_1_, *y*_2_, ..., *y*_*m*_} of *X*, let *c*(*Y*) denote the centroid of *Y *and let its variance be

VAR(Y)=1m∑i=1m∑j=1d(yi,j−c(Y)j)2.     (1)
 MathType@MTEF@5@5@+=feaafiart1ev1aaatCvAUfKttLearuWrP9MDH5MBPbIqV92AaeXatLxBI9gBaebbnrfifHhDYfgasaacH8akY=wiFfYdH8Gipec8Eeeu0xXdbba9frFj0=OqFfea0dXdd9vqai=hGuQ8kuc9pgc9s8qqaq=dirpe0xb9q8qiLsFr0=vr0=vr0dc8meaabaqaciGacaGaaeqabaqabeGadaaakeaacqWGwbGvcqWGbbqqcqWGsbGucqGGOaakcqWGzbqwcqGGPaqkcqGH9aqpdaWcaaqaaiabigdaXaqaaiabd2gaTbaadaaeWbqaamaaqahabaGaeiikaGIaemyEaK3aaSbaaSqaaiabdMgaPjabcYcaSiabdQgaQbqabaGccqGHsislcqWGJbWycqGGOaakcqWGzbqwcqGGPaqkdaWgaaWcbaGaemOAaOgabeaakiabcMcaPmaaCaaaleqabaGaeGOmaidaaOGaeiOla4caleaacqWGQbGAcqGH9aqpcqaIXaqmaeaacqWGKbaza0GaeyyeIuoaaSqaaiabdMgaPjabg2da9iabigdaXaqaaiabd2gaTbqdcqGHris5aOGaaCzcaiaaxMaacqGGOaakcqaIXaqmcqGGPaqkaaa@5801@

Given an integer *k*, we are interested in finding a partition P
 MathType@MTEF@5@5@+=feaafiart1ev1aaatCvAUfKttLearuWrP9MDH5MBPbIqV92AaeXatLxBI9gBamXvP5wqSXMqHnxAJn0BKvguHDwzZbqegm0B1jxALjhiov2DaebbnrfifHhDYfgasaacH8akY=wiFfYdH8Gipec8Eeeu0xXdbba9frFj0=OqFfea0dXdd9vqai=hGuQ8kuc9pgc9s8qqaq=dirpe0xb9q8qiLsFr0=vr0=vr0dc8meaabaqaciaacaGaaeqabaWaaeGaeaaakeaaimaacaWFqbaaaa@396B@ of *X *into *k *classes *C*_0_, *C*_1 _..., *C*_*k*-1 _so that the total internal variance

VAR(P)=∑i=0k−1VAR(Ci)     (2)
 MathType@MTEF@5@5@+=feaafiart1ev1aaatCvAUfKttLearuWrP9MDH5MBPbIqV92AaeXatLxBI9gBamXvP5wqSXMqHnxAJn0BKvguHDwzZbqegm0B1jxALjhiov2DaebbnrfifHhDYfgasaacH8akY=wiFfYdH8Gipec8Eeeu0xXdbba9frFj0=OqFfea0dXdd9vqai=hGuQ8kuc9pgc9s8qqaq=dirpe0xb9q8qiLsFr0=vr0=vr0dc8meaabaqaciaacaGaaeqabaWaaeGaeaaakeaacqWGwbGvcqWGbbqqcqWGsbGucqGGOaakimaacaWFqbGaeiykaKIaeyypa0ZaaabCaeaacqWGwbGvcqWGbbqqcqWGsbGucqGGOaakcqWGdbWqdaWgaaWcbaGaemyAaKgabeaakiabcMcaPaWcbaGaemyAaKMaeyypa0JaeGymaedabaGaem4AaSMaeyOeI0IaeGymaedaniabggHiLdGccaWLjaGaaCzcaiabcIcaOiabikdaYiabcMcaPaaa@5410@

is minimized. *GenClust *provides a feasible solution to the posed optimization problem, and experiments show its convergence to a local optimum.

### The algorithm GenClust

*GenClust *proceeds in stages, producing a sequence of partitions Pi
 MathType@MTEF@5@5@+=feaafiart1ev1aaatCvAUfKttLearuWrP9MDH5MBPbIqV92AaeXatLxBI9gBamXvP5wqSXMqHnxAJn0BKvguHDwzZbqegm0B1jxALjhiov2DaebbnrfifHhDYfgasaacH8akY=wiFfYdH8Gipec8Eeeu0xXdbba9frFj0=OqFfea0dXdd9vqai=hGuQ8kuc9pgc9s8qqaq=dirpe0xb9q8qiLsFr0=vr0=vr0dc8meaabaqaciaacaGaaeqabaWaaeGaeaaakeaaimaacaWFqbWaaSbaaSqaaiabdMgaPbqabaaaaa@3AF2@, each consisting of *k *classes, until a halting condition is met. Let *α *= (*x*, *λ*) be an *individual*, *x *∈ *X *and 0 ≤ *λ *<*k*. A partition Pi
 MathType@MTEF@5@5@+=feaafiart1ev1aaatCvAUfKttLearuWrP9MDH5MBPbIqV92AaeXatLxBI9gBamXvP5wqSXMqHnxAJn0BKvguHDwzZbqegm0B1jxALjhiov2DaebbnrfifHhDYfgasaacH8akY=wiFfYdH8Gipec8Eeeu0xXdbba9frFj0=OqFfea0dXdd9vqai=hGuQ8kuc9pgc9s8qqaq=dirpe0xb9q8qiLsFr0=vr0=vr0dc8meaabaqaciaacaGaaeqabaWaaeGaeaaakeaaimaacaWFqbWaaSbaaSqaaiabdMgaPbqabaaaaa@3AF2@ is best seen as a collection of individuals arranged in any order, i.e., a population. Only at the end, *GenClust *assembles elements according to cluster number. Following the evolutionary computational paradigm, a population evolves by means of genetic operators, i.e., cross-over, mutation and selection, resulting in a random walk in cluster space, where the fitness function gives a drift to the process towards a local optimum.

The internal data representation and coding is crucial to *GenClust*. The elements of *X *are stored into an *n *× *d *matrix, and the row *r*(*x*), corresponding to *x*, is the internal name of *x*. We also keep the inverse mapping *r*^-1^(*i*) = *x*, 0 ≤ *i *<*n *- 1. A partition P
 MathType@MTEF@5@5@+=feaafiart1ev1aaatCvAUfKttLearuWrP9MDH5MBPbIqV92AaeXatLxBI9gBamXvP5wqSXMqHnxAJn0BKvguHDwzZbqegm0B1jxALjhiov2DaebbnrfifHhDYfgasaacH8akY=wiFfYdH8Gipec8Eeeu0xXdbba9frFj0=OqFfea0dXdd9vqai=hGuQ8kuc9pgc9s8qqaq=dirpe0xb9q8qiLsFr0=vr0=vr0dc8meaabaqaciaacaGaaeqabaWaaeGaeaaakeaaimaacaWFqbaaaa@396B@ of *X *is encoded with a list of *n *32-bit strings, each representing an individual (*x*, *λ*). That individual is encoded, one-to-many, by arbitrarily choosing a string *s *from a set of 32-bit strings, as follows. The least significant 8 bits of *s *give a "representation" of *λ *and the remaining ones a "representation" of *r*(*x*). If *r*(*x*) is in [0, *n *- 2], the binary encoding of any integer in [i∗⌊224n⌋,(i+1)∗⌊224n⌋−1]
 MathType@MTEF@5@5@+=feaafiart1ev1aaatCvAUfKttLearuWrP9MDH5MBPbIqV92AaeXatLxBI9gBaebbnrfifHhDYfgasaacH8akY=wiFfYdH8Gipec8Eeeu0xXdbba9frFj0=OqFfea0dXdd9vqai=hGuQ8kuc9pgc9s8qqaq=dirpe0xb9q8qiLsFr0=vr0=vr0dc8meaabaqaciGacaGaaeqabaqabeGadaaakeaadaWadaqaaiabdMgaPjabgEHiQmaagmaabaWaaSaaaeaacqaIYaGmdaahaaWcbeqaaiabikdaYiabisda0aaaaOqaaiabd6gaUbaaaiaawcp+caGL7JpacqGGSaalcqGGOaakcqWGPbqAcqGHRaWkcqaIXaqmcqGGPaqkcqGHxiIkdaGbdaqaamaalaaabaGaeGOmaiZaaWbaaSqabeaacqaIYaGmcqaI0aanaaaakeaacqWGUbGBaaaacaGLWJVaay5+4dGaeyOeI0IaeGymaedacaGLBbGaayzxaaaaaa@4CB1@ will do. Otherwise, the binary encoding of any integer in [n⌊224n⌋,224−1]
 MathType@MTEF@5@5@+=feaafiart1ev1aaatCvAUfKttLearuWrP9MDH5MBPbIqV92AaeXatLxBI9gBaebbnrfifHhDYfgasaacH8akY=wiFfYdH8Gipec8Eeeu0xXdbba9frFj0=OqFfea0dXdd9vqai=hGuQ8kuc9pgc9s8qqaq=dirpe0xb9q8qiLsFr0=vr0=vr0dc8meaabaqaciGacaGaaeqabaqabeGadaaakeaadaWadaqaaiabd6gaUnaagmaabaWaaSaaaeaacqaIYaGmdaahaaWcbeqaaiabikdaYiabisda0aaaaOqaaiabd6gaUbaaaiaawcp+caGL7JpacqGGSaalcqaIYaGmdaahaaWcbeqaaiabikdaYiabisda0aaakiabgkHiTiabigdaXaGaay5waiaaw2faaaaa@3F71@ will do. Analogous rules apply to *λ*, except that 2^24 ^and *n *are replaced by 2^8 ^and *k*, respectively. Given any 32-bit string, we can recover in a constant number of operations the unique (*r*(*x*), *λ*) of which it can be an encoding, and therefore (*x*, *λ*) (via the inverse mapping r^-1^). The straightforward details are omitted. In what follows, *D*(*s*) returns (*r*(*x*), *λ*), with *D*_1_(*s*) = *r*(*x*) and *D*_2_(*s*) = *λ*, *x *∈ *X *and 0 ≤ *λ *<*k*. The chosen encoding is compact, easy to handle, and allows up to 256 classes and data sets of size up to 16,793,604 elements, values adequate for real applications.

The initial partition P0
 MathType@MTEF@5@5@+=feaafiart1ev1aaatCvAUfKttLearuWrP9MDH5MBPbIqV92AaeXatLxBI9gBamXvP5wqSXMqHnxAJn0BKvguHDwzZbqegm0B1jxALjhiov2DaebbnrfifHhDYfgasaacH8akY=wiFfYdH8Gipec8Eeeu0xXdbba9frFj0=OqFfea0dXdd9vqai=hGuQ8kuc9pgc9s8qqaq=dirpe0xb9q8qiLsFr0=vr0=vr0dc8meaabaqaciaacaGaaeqabaWaaeGaeaaakeaaimaacaWFqbWaaSbaaSqaaiabicdaWaqabaaaaa@3A85@ can be computed by either randomly partitioning the elements of *X *into *k *classes or by using a user specified partition of the elements of *X*, such as the one produced by yet another clustering algorithm.

The heart of *GenClust *is the transition in cluster space from Pi
 MathType@MTEF@5@5@+=feaafiart1ev1aaatCvAUfKttLearuWrP9MDH5MBPbIqV92AaeXatLxBI9gBamXvP5wqSXMqHnxAJn0BKvguHDwzZbqegm0B1jxALjhiov2DaebbnrfifHhDYfgasaacH8akY=wiFfYdH8Gipec8Eeeu0xXdbba9frFj0=OqFfea0dXdd9vqai=hGuQ8kuc9pgc9s8qqaq=dirpe0xb9q8qiLsFr0=vr0=vr0dc8meaabaqaciaacaGaaeqabaWaaeGaeaaakeaaimaacaWFqbWaaSbaaSqaaiabdMgaPbqabaaaaa@3AF2@ to Pi+1
 MathType@MTEF@5@5@+=feaafiart1ev1aaatCvAUfKttLearuWrP9MDH5MBPbIqV92AaeXatLxBI9gBamXvP5wqSXMqHnxAJn0BKvguHDwzZbqegm0B1jxALjhiov2DaebbnrfifHhDYfgasaacH8akY=wiFfYdH8Gipec8Eeeu0xXdbba9frFj0=OqFfea0dXdd9vqai=hGuQ8kuc9pgc9s8qqaq=dirpe0xb9q8qiLsFr0=vr0=vr0dc8meaabaqaciaacaGaaeqabaWaaeGaeaaakeaaimaacaWFqbWaaSbaaSqaaiabdMgaPjabgUcaRiabigdaXaqabaaaaa@3CC4@, *i *≥ 0. This is accomplished by a proper manipulation of the 32-bit strings in the list *L*_*i *_= (*s*_0_, *s*_1_, ..., *s*_*n*-1_) encoding Pi
 MathType@MTEF@5@5@+=feaafiart1ev1aaatCvAUfKttLearuWrP9MDH5MBPbIqV92AaeXatLxBI9gBamXvP5wqSXMqHnxAJn0BKvguHDwzZbqegm0B1jxALjhiov2DaebbnrfifHhDYfgasaacH8akY=wiFfYdH8Gipec8Eeeu0xXdbba9frFj0=OqFfea0dXdd9vqai=hGuQ8kuc9pgc9s8qqaq=dirpe0xb9q8qiLsFr0=vr0=vr0dc8meaabaqaciaacaGaaeqabaWaaeGaeaaakeaaimaacaWFqbWaaSbaaSqaaiabdMgaPbqabaaaaa@3AF2@. Assume that *L*_*i *_is sorted according to the internal representation of the elements; i.e., *D*_1_(*s*_*p*_) <*D*_1_(*s*_*j*_), *p *<*j*. The following steps are applied in order.

#### Cross-over

The objective is to produce a list *L*_*temp *_of new binary strings by properly recombining the ones in *L*_*i*_. For each string *s*_*j*_, 0 ≤ *j *<*n*, the standard one point cross-over operation is performed [16], with probability 0.9. The second string is chosen at random from the ones in *L*_*i *_- {*s*_*j*_}. The cross-over operation generates two new strings that are appended to *L*_*temp*_. At the end, *L*_*temp *_is a list of *m *≤ *n *32-bits strings. Notice that, because of the encoding and decoding process we are using, the recombined string will still represent a pair (*r*(*x*), *λ*), with 0 ≤ *r*(*x*) <*n *and 0 ≤ *λ *<*k*.

#### First selection

Notice that while each string in *L*_*i *_corresponds to exactly one element *x *∈ *X *and vice versa, that is no longer true for the concatenated lists *L*_*i *_○ *L*_*temp*_. We eliminate duplicates by keeping only the rightmost string *s *in L_*i *_○ *L*_*temp *_such that *D*_1_(*s*) = *j*, for *j *= 0, ..., *n *- 1. Denote the result by *L'*.

#### One-bit mutation

*L' *is an encoding of a partition related to Pi
 MathType@MTEF@5@5@+=feaafiart1ev1aaatCvAUfKttLearuWrP9MDH5MBPbIqV92AaeXatLxBI9gBamXvP5wqSXMqHnxAJn0BKvguHDwzZbqegm0B1jxALjhiov2DaebbnrfifHhDYfgasaacH8akY=wiFfYdH8Gipec8Eeeu0xXdbba9frFj0=OqFfea0dXdd9vqai=hGuQ8kuc9pgc9s8qqaq=dirpe0xb9q8qiLsFr0=vr0=vr0dc8meaabaqaciaacaGaaeqabaWaaeGaeaaakeaaimaacaWFqbWaaSbaaSqaaiabdMgaPbqabaaaaa@3AF2@. In order to climb out of local minima, it is perturbed as follows. For *j *= 0, ..., *n *- 1, a one-bit mutation is applied to s′j
 MathType@MTEF@5@5@+=feaafiart1ev1aaatCvAUfKttLearuWrP9MDH5MBPbIqV92AaeXatLxBI9gBaebbnrfifHhDYfgasaacH8akY=wiFfYdH8Gipec8Eeeu0xXdbba9frFj0=OqFfea0dXdd9vqai=hGuQ8kuc9pgc9s8qqaq=dirpe0xb9q8qiLsFr0=vr0=vr0dc8meaabaqaciGacaGaaeqabaqabeGadaaakeaacuWGZbWCgaqbamaaBaaaleaacqWGQbGAaeqaaaaa@2FB2@ ∈ *L' *with probability 0.01, resulting in a string *s*. There are several possible outcomes. The mutation is silent, i.e., *D*(s′j
 MathType@MTEF@5@5@+=feaafiart1ev1aaatCvAUfKttLearuWrP9MDH5MBPbIqV92AaeXatLxBI9gBaebbnrfifHhDYfgasaacH8akY=wiFfYdH8Gipec8Eeeu0xXdbba9frFj0=OqFfea0dXdd9vqai=hGuQ8kuc9pgc9s8qqaq=dirpe0xb9q8qiLsFr0=vr0=vr0dc8meaabaqaciGacaGaaeqabaqabeGadaaakeaacuWGZbWCgaqbamaaBaaaleaacqWGQbGAaeqaaaaa@2FB2@) = *D*(*s*). No action is taken. It affects the cluster membership of *D*_1_(s′j
 MathType@MTEF@5@5@+=feaafiart1ev1aaatCvAUfKttLearuWrP9MDH5MBPbIqV92AaeXatLxBI9gBaebbnrfifHhDYfgasaacH8akY=wiFfYdH8Gipec8Eeeu0xXdbba9frFj0=OqFfea0dXdd9vqai=hGuQ8kuc9pgc9s8qqaq=dirpe0xb9q8qiLsFr0=vr0=vr0dc8meaabaqaciGacaGaaeqabaqabeGadaaakeaacuWGZbWCgaqbamaaBaaaleaacqWGQbGAaeqaaaaa@2FB2@), i.e., *D*_1_(s′j
 MathType@MTEF@5@5@+=feaafiart1ev1aaatCvAUfKttLearuWrP9MDH5MBPbIqV92AaeXatLxBI9gBaebbnrfifHhDYfgasaacH8akY=wiFfYdH8Gipec8Eeeu0xXdbba9frFj0=OqFfea0dXdd9vqai=hGuQ8kuc9pgc9s8qqaq=dirpe0xb9q8qiLsFr0=vr0=vr0dc8meaabaqaciGacaGaaeqabaqabeGadaaakeaacuWGZbWCgaqbamaaBaaaleaacqWGQbGAaeqaaaaa@2FB2@) = *D*_1_(*s*) but *D*_2_(s′j
 MathType@MTEF@5@5@+=feaafiart1ev1aaatCvAUfKttLearuWrP9MDH5MBPbIqV92AaeXatLxBI9gBaebbnrfifHhDYfgasaacH8akY=wiFfYdH8Gipec8Eeeu0xXdbba9frFj0=OqFfea0dXdd9vqai=hGuQ8kuc9pgc9s8qqaq=dirpe0xb9q8qiLsFr0=vr0=vr0dc8meaabaqaciGacaGaaeqabaqabeGadaaakeaacuWGZbWCgaqbamaaBaaaleaacqWGQbGAaeqaaaaa@2FB2@) ≠ *D*_2_(*s*), or it causes a collision, i.e., there exists an s′p
 MathType@MTEF@5@5@+=feaafiart1ev1aaatCvAUfKttLearuWrP9MDH5MBPbIqV92AaeXatLxBI9gBaebbnrfifHhDYfgasaacH8akY=wiFfYdH8Gipec8Eeeu0xXdbba9frFj0=OqFfea0dXdd9vqai=hGuQ8kuc9pgc9s8qqaq=dirpe0xb9q8qiLsFr0=vr0=vr0dc8meaabaqaciGacaGaaeqabaqabeGadaaakeaacuWGZbWCgaqbamaaBaaaleaacqWGWbaCaeqaaaaa@2FBE@ in *L'*, *p *≠ *j*, such that *D*_1_(*s*) = *D*_1_(s′p
 MathType@MTEF@5@5@+=feaafiart1ev1aaatCvAUfKttLearuWrP9MDH5MBPbIqV92AaeXatLxBI9gBaebbnrfifHhDYfgasaacH8akY=wiFfYdH8Gipec8Eeeu0xXdbba9frFj0=OqFfea0dXdd9vqai=hGuQ8kuc9pgc9s8qqaq=dirpe0xb9q8qiLsFr0=vr0=vr0dc8meaabaqaciGacaGaaeqabaqabeGadaaakeaacuWGZbWCgaqbamaaBaaaleaacqWGWbaCaeqaaaaa@2FBE@). Then, *s *replaces s′j
 MathType@MTEF@5@5@+=feaafiart1ev1aaatCvAUfKttLearuWrP9MDH5MBPbIqV92AaeXatLxBI9gBaebbnrfifHhDYfgasaacH8akY=wiFfYdH8Gipec8Eeeu0xXdbba9frFj0=OqFfea0dXdd9vqai=hGuQ8kuc9pgc9s8qqaq=dirpe0xb9q8qiLsFr0=vr0=vr0dc8meaabaqaciGacaGaaeqabaqabeGadaaakeaacuWGZbWCgaqbamaaBaaaleaacqWGQbGAaeqaaaaa@2FB2@.

#### Second selection

We have now two lists *L*_*i *_and *L' *of *n *32-bit strings, representing the encoding of Pi
 MathType@MTEF@5@5@+=feaafiart1ev1aaatCvAUfKttLearuWrP9MDH5MBPbIqV92AaeXatLxBI9gBamXvP5wqSXMqHnxAJn0BKvguHDwzZbqegm0B1jxALjhiov2DaebbnrfifHhDYfgasaacH8akY=wiFfYdH8Gipec8Eeeu0xXdbba9frFj0=OqFfea0dXdd9vqai=hGuQ8kuc9pgc9s8qqaq=dirpe0xb9q8qiLsFr0=vr0=vr0dc8meaabaqaciaacaGaaeqabaWaaeGaeaaakeaaimaacaWFqbWaaSbaaSqaaiabdMgaPbqabaaaaa@3AF2@ and P′i
 MathType@MTEF@5@5@+=feaafiart1ev1aaatCvAUfKttLearuWrP9MDH5MBPbIqV92AaeXatLxBI9gBamXvP5wqSXMqHnxAJn0BKvguHDwzZbqegm0B1jxALjhiov2DaebbnrfifHhDYfgasaacH8akY=wiFfYdH8Gipec8Eeeu0xXdbba9frFj0=OqFfea0dXdd9vqai=hGuQ8kuc9pgc9s8qqaq=dirpe0xb9q8qiLsFr0=vr0=vr0dc8meaabaqaciaacaGaaeqabaWaaeGaeaaakeaaimaaceWFqbGbauaadaWgaaWcbaGaemyAaKgabeaaaaa@3AFE@ where this latter one is possibly a new partition. Let *L' *be sorted according to the internal representation of the elements, i.e., *D*_1_(s′p
 MathType@MTEF@5@5@+=feaafiart1ev1aaatCvAUfKttLearuWrP9MDH5MBPbIqV92AaeXatLxBI9gBaebbnrfifHhDYfgasaacH8akY=wiFfYdH8Gipec8Eeeu0xXdbba9frFj0=OqFfea0dXdd9vqai=hGuQ8kuc9pgc9s8qqaq=dirpe0xb9q8qiLsFr0=vr0=vr0dc8meaabaqaciGacaGaaeqabaqabeGadaaakeaacuWGZbWCgaqbamaaBaaaleaacqWGWbaCaeqaaaaa@2FBE@) <*D*_1_(s′j
 MathType@MTEF@5@5@+=feaafiart1ev1aaatCvAUfKttLearuWrP9MDH5MBPbIqV92AaeXatLxBI9gBaebbnrfifHhDYfgasaacH8akY=wiFfYdH8Gipec8Eeeu0xXdbba9frFj0=OqFfea0dXdd9vqai=hGuQ8kuc9pgc9s8qqaq=dirpe0xb9q8qiLsFr0=vr0=vr0dc8meaabaqaciGacaGaaeqabaqabeGadaaakeaacuWGZbWCgaqbamaaBaaaleaacqWGQbGAaeqaaaaa@2FB2@), *p *<*j*. The encoding *L*_*i*+1 _= {*c*_0_, ..., *c*_*n*-1_} of Pi+1
 MathType@MTEF@5@5@+=feaafiart1ev1aaatCvAUfKttLearuWrP9MDH5MBPbIqV92AaeXatLxBI9gBamXvP5wqSXMqHnxAJn0BKvguHDwzZbqegm0B1jxALjhiov2DaebbnrfifHhDYfgasaacH8akY=wiFfYdH8Gipec8Eeeu0xXdbba9frFj0=OqFfea0dXdd9vqai=hGuQ8kuc9pgc9s8qqaq=dirpe0xb9q8qiLsFr0=vr0=vr0dc8meaabaqaciaacaGaaeqabaWaaeGaeaaakeaaimaacaWFqbWaaSbaaSqaaiabdMgaPjabgUcaRiabigdaXaqabaaaaa@3CC4@ is obtained via the following selection process:

cr={s′rif f(D(s′r))<f(D(sr))srotherwise
 MathType@MTEF@5@5@+=feaafiart1ev1aaatCvAUfKttLearuWrP9MDH5MBPbIqV92AaeXatLxBI9gBaebbnrfifHhDYfgasaacH8akY=wiFfYdH8Gipec8Eeeu0xXdbba9frFj0=OqFfea0dXdd9vqai=hGuQ8kuc9pgc9s8qqaq=dirpe0xb9q8qiLsFr0=vr0=vr0dc8meaabaqaciGacaGaaeqabaqabeGadaaakeaacqWGJbWydaWgaaWcbaGaemOCaihabeaakiabg2da9maaceaabaqbaeaabiGaaaqaaiqbdohaZzaafaWaaSbaaSqaaiabdkhaYbqabaaakeaacqWGPbqAcqWGMbGzcaaMc8UaemOzayMaeiikaGIaemiraqKaeiikaGIafm4CamNbauaadaWgaaWcbaGaemOCaihabeaakiabcMcaPiabcMcaPiabgYda8iabdAgaMjabcIcaOiabdseaejabcIcaOiabdohaZnaaBaaaleaacqWGYbGCaeqaaOGaeiykaKIaeiykaKcabaGaem4Cam3aaSbaaSqaaiabdkhaYbqabaaakeaacqqGVbWBcqqG0baDcqqGObaAcqqGLbqzcqqGYbGCcqqG3bWDcqqGPbqAcqqGZbWCcqqGLbqzaaaacaGL7baaaaa@5B75@

*r *= 0, ..., n - 1 and where

f((x,λ))=1d∑j=1d(xj−c(Cλ)j)2max⁡(xj,c(Cλ)j))2     (3)
 MathType@MTEF@5@5@+=feaafiart1ev1aaatCvAUfKttLearuWrP9MDH5MBPbIqV92AaeXatLxBI9gBaebbnrfifHhDYfgasaacH8akY=wiFfYdH8Gipec8Eeeu0xXdbba9frFj0=OqFfea0dXdd9vqai=hGuQ8kuc9pgc9s8qqaq=dirpe0xb9q8qiLsFr0=vr0=vr0dc8meaabaqaciGacaGaaeqabaqabeGadaaakeaacqWGMbGzcqGGOaakcqGGOaakcqWG4baEcqGGSaalcqaH7oaBcqGGPaqkcqGGPaqkcqGH9aqpdaGcaaqaamaalaaabaGaeGymaedabaGaemizaqgaamaaqahabaWaaSaaaeaacqGGOaakcqWG4baEdaWgaaWcbaGaemOAaOgabeaakiabgkHiTiabdogaJjabcIcaOiabdoeadnaaBaaaleaacqaH7oaBaeqaaOGaeiykaKYaaSbaaSqaaiabdQgaQbqabaGccqGGPaqkdaahaaWcbeqaaiabikdaYaaaaOqaaiGbc2gaTjabcggaHjabcIha4jabcIcaOiabdIha4naaBaaaleaacqWGQbGAaeqaaOGaeiilaWIaem4yamMaeiikaGIaem4qam0aaSbaaSqaaiabeU7aSbqabaGccqGGPaqkdaWgaaWcbaGaemOAaOgabeaakiabcMcaPiabcMcaPmaaCaaaleqabaGaeGOmaidaaaaaaeaacqWGQbGAcqGH9aqpcqaIXaqmaeaacqWGKbaza0GaeyyeIuoaaSqabaGccaWLjaGaaCzcaiabcIcaOiabiodaZiabcMcaPaaa@6570@

is the *fitness function *of individual (*x*, *λ*) in a generic partition P
 MathType@MTEF@5@5@+=feaafiart1ev1aaatCvAUfKttLearuWrP9MDH5MBPbIqV92AaeXatLxBI9gBamXvP5wqSXMqHnxAJn0BKvguHDwzZbqegm0B1jxALjhiov2DaebbnrfifHhDYfgasaacH8akY=wiFfYdH8Gipec8Eeeu0xXdbba9frFj0=OqFfea0dXdd9vqai=hGuQ8kuc9pgc9s8qqaq=dirpe0xb9q8qiLsFr0=vr0=vr0dc8meaabaqaciaacaGaaeqabaWaaeGaeaaakeaaimaacaWFqbaaaa@396B@, and *C*_*λ *_is cluster number *λ *in that partition. That is, *f*(*D*(s′r
 MathType@MTEF@5@5@+=feaafiart1ev1aaatCvAUfKttLearuWrP9MDH5MBPbIqV92AaeXatLxBI9gBaebbnrfifHhDYfgasaacH8akY=wiFfYdH8Gipec8Eeeu0xXdbba9frFj0=OqFfea0dXdd9vqai=hGuQ8kuc9pgc9s8qqaq=dirpe0xb9q8qiLsFr0=vr0=vr0dc8meaabaqaciGacaGaaeqabaqabeGadaaakeaacuWGZbWCgaqbamaaBaaaleaacqWGYbGCaeqaaaaa@2FC2@)) refers to the partition encoded by *L' *and *f*(*D*(*s*_*r*_)) to the one encoded by *L*_*i*_.

There are several types of halting criteria that can be used for *GenClust*. We have considered one in which the algorithm is given a user-specified number of iterations, i.e., number of partitions Pi
 MathType@MTEF@5@5@+=feaafiart1ev1aaatCvAUfKttLearuWrP9MDH5MBPbIqV92AaeXatLxBI9gBamXvP5wqSXMqHnxAJn0BKvguHDwzZbqegm0B1jxALjhiov2DaebbnrfifHhDYfgasaacH8akY=wiFfYdH8Gipec8Eeeu0xXdbba9frFj0=OqFfea0dXdd9vqai=hGuQ8kuc9pgc9s8qqaq=dirpe0xb9q8qiLsFr0=vr0=vr0dc8meaabaqaciaacaGaaeqabaWaaeGaeaaakeaaimaacaWFqbWaaSbaaSqaaiabdMgaPbqabaaaaa@3AF2@ to produce. At each iteration, apart from the current partition, it also keeps track of the partition corresponding to the best internal variance seen over the iterations performed so far. Another user-specified parameter indicates whether, at the end of the iterations, the algorithm must output the last partition or the one corresponding to the minimum internal variance seen during its execution. We refer to those partitions as Plast
 MathType@MTEF@5@5@+=feaafiart1ev1aaatCvAUfKttLearuWrP9MDH5MBPbIqV92AaeXatLxBI9gBamXvP5wqSXMqHnxAJn0BKvguHDwzZbqegm0B1jxALjhiov2DaebbnrfifHhDYfgasaacH8akY=wiFfYdH8Gipec8Eeeu0xXdbba9frFj0=OqFfea0dXdd9vqai=hGuQ8kuc9pgc9s8qqaq=dirpe0xb9q8qiLsFr0=vr0=vr0dc8meaabaqaciaacaGaaeqabaWaaeGaeaaakeaaimaacaWFqbWaaSbaaSqaaiabdYgaSjabdggaHjabdohaZjabdsha0bqabaaaaa@3F23@ and Pbest
 MathType@MTEF@5@5@+=feaafiart1ev1aaatCvAUfKttLearuWrP9MDH5MBPbIqV92AaeXatLxBI9gBamXvP5wqSXMqHnxAJn0BKvguHDwzZbqegm0B1jxALjhiov2DaebbnrfifHhDYfgasaacH8akY=wiFfYdH8Gipec8Eeeu0xXdbba9frFj0=OqFfea0dXdd9vqai=hGuQ8kuc9pgc9s8qqaq=dirpe0xb9q8qiLsFr0=vr0=vr0dc8meaabaqaciaacaGaaeqabaWaaeGaeaaakeaaimaacaWFqbWaaSbaaSqaaiabdkgaIjabdwgaLjabdohaZjabdsha0bqabaaaaa@3F17@, respectively. The rationale behind the described mode of operation is to allow *GenClust *to climb out of local optima. Since the number of iterations must be determined experimentally, the algorithm outputs also two auxiliary files: *variance*, reporting the values of internal variance, and *best*, internal variance for each iteration. This point is related to the convergence of *GenClust *to a local optimum and is discussed in the Experiments subsection.

We point out that the inherent freedom of the one-to-many mapping of individuals to binary strings, which we have used, provides enough flexibility so that *GenClust *can work on one single partition, allowing it to change. This should be contrasted with other existing clustering algorithms based on the genetic paradigm, since at each stage, they typically maintain a family of partitions [14,15]. This results in higher computational demand when going from one iteration to the next.

Since *GenClust *needs in input the number *k *of clusters, it must be used in conjunction with a methodology that guides in the estimation of the real number of clusters in a data set and also evaluates the quality of clustering solutions. We have chosen FOM for our experiments, since it has had great impact on the scientific literature in this area. Valid alternatives are described in [8,9], where additional references to the literature are also given. Data reduction techniques, such as filtering [20] and principal component analysis may also be of help in those circumstances.

## Results and discussion

### Experimental methodology

We have chosen data sets for which a biological meaningful partition into classes is known in the literature: e.g., biologically distinct functional classes. We refer to that partition as the *true solution*. We have also chosen a suite of algorithms, *Average Link *among the *Hierarchical Methods *[5], *K-means *[5], *Cast, Click *against which we compare the performance of *GenClust*, established by means of external and internal criteria. The external criteria measure how well a clustering solution computed by an algorithm agrees with the *true solution *for a given data set. Among the many available [11], we have chosen the adjusted Rand index [21], a flexible index allowing comparison among partitions with different numbers of classes and also recommended in the statistics and classification literature [22,23]. When the true solution is not known, the internal criteria must give a reliable indication of how well a partitioning solution produced by an algorithm captures the inherent separation of the data into clusters, i.e., how many clusters are really present in the data. We have chosen FOM for our experiments.

#### Data sets

**RCNS**. The data set is obtained by reverse transcription coupled PCR to study the expression levels of 112 genes during rat central nervous system development over 9 time points [24]. That results in a 112*x*9 data matrix. It was studied by Wen et al. [25] to obtain a division of the genes into 6 classes, four of which are composed of biologically functionally related genes. This division is assumed to be the *true solution*. Before the analysis, Wen et al. performed two transformations on the data for each gene: (a) each row is divided by its maximum value; (b) to capture the temporal nature of the data, the difference between the values of two consecutive data points is added as an extra data point. Therefore, the final data set consists of a 112*x*17 data matrix, which is the input to our algorithms. We point out that the second transformation has the effect to enhance the similarity between genes with closely parallel, but offset, expression patterns.

**YCC**. The data set is part of that studied by Spellman et al. [26] and has been used by Sharan et al. for validation of their clustering algorithm *Click*. The complete data set contains the expression levels of roughly 6000 yeast ORFs over 79 conditions. The analysis by Spellman et al. identified 800 genes that are cell cycle regulated. In order to demonstrate the validity of *Click*, Sharan et al. extracted 698 out of those 800 genes, over 72 conditions, by eliminating all genes that had at least three missing entries. Additional details on that "extraction process" can be found in [17]. The resulting 698*x*72 data matrix is standardized (i.e., for each row, the entries are scaled so that the mean is zero and the variance is one) and used for our experiments. The *true solution *is given by the partition of the 698 extracted genes according to the five functional classes they belong to in the classification by Spellman et al.

**RYCC**. This data set originates in the one by Cho et al. [27] for the study of yeast cell cycle regulated genes and has been created and used by Ka Yee Yeung for her study of FOM in her doctoral dissertation [11]. Ka Yee Yeung extracted 384 genes from the yeast cell cycle data set in Cho et al. to obtain a 384*x*17 data expression matrix. The details of the extraction process are in [28]. That matrix is then standardized as in Tamayo et al. [20]. That is, the data matrix is divided in two contiguous pieces and each piece is standardized separately. We use that standardized data set for our experiments and assume as the *true solution *the same as in the dissertation by Ka Yee Yeung. It is to be pointed out that each gene in the **RYCC **data set appears also in the **YCC **data set. However, the dimensionality of the two data sets is quite different, and this may cause algorithms to behave differently. Moreover, **RYCC **is also useful for a qualitative comparison of our results with the ones in the doctoral dissertation by Ka Yee Yeung.

**PBM**. The data set was used by Hartuv et al. [29] to test their clustering algorithm. It contains 2329 cDNAs with a fingerprint of 139 oligos. This gives a 2329*x*139 data matrix. Each row corresponds to a gene, but different rows may correspond to the same gene. The true solution consists of a division of the rows in 18 classes, i.e., the data set consists of 18 genes.

**RPBM**. Since FOM was too time demanding to complete its execution on the data set by Hartuv et al., we have reduced the data in order to get an indication of the number of clusters in the data set. We have randomly picked 10% of the cDNAs in each of the 18 original classes. Whenever that percentage is less than one, we have retained the entire class. The result is a 235*x*139 data matrix, and the *true solution *is readily obtained from that of **PBM**. Data sets are provided as supplementary material [30].

#### Algorithms

*Average Link *has been implemented, among the hierarchical methods. Following prior work [11,12], a dendogram is built bottom-up until one obtains *k *subtrees, for a user-specified parameter *k*. Then, *k *clusters are obtained by assuming that the genes at the leaves of each subtree form a distinct cluster. We have also implemented *GenClust *and *K-means*. Both algorithms take as input a parameter *k *and return *k *clusters. They can either start with a randomly generated initial partition of the genes in *k *classes, or they can take as input a user-specified partition of the elements, for instance the output of yet another clustering algorithm. For our experiments, we have chosen the output of *Average Link *in this second case. In what follows, the type of initial partition chosen for those two algorithms appear as a suffix, i.e., *K-means-Random *means that the initial partition has been generated at random. Moreover, since *GenClust *can output one of two partitions, i.e., Plast
 MathType@MTEF@5@5@+=feaafiart1ev1aaatCvAUfKttLearuWrP9MDH5MBPbIqV92AaeXatLxBI9gBamXvP5wqSXMqHnxAJn0BKvguHDwzZbqegm0B1jxALjhiov2DaebbnrfifHhDYfgasaacH8akY=wiFfYdH8Gipec8Eeeu0xXdbba9frFj0=OqFfea0dXdd9vqai=hGuQ8kuc9pgc9s8qqaq=dirpe0xb9q8qiLsFr0=vr0=vr0dc8meaabaqaciaacaGaaeqabaWaaeGaeaaakeaaimaacaWFqbWaaSbaaSqaaiabdYgaSjabdggaHjabdohaZjabdsha0bqabaaaaa@3F23@ or Pbest
 MathType@MTEF@5@5@+=feaafiart1ev1aaatCvAUfKttLearuWrP9MDH5MBPbIqV92AaeXatLxBI9gBamXvP5wqSXMqHnxAJn0BKvguHDwzZbqegm0B1jxALjhiov2DaebbnrfifHhDYfgasaacH8akY=wiFfYdH8Gipec8Eeeu0xXdbba9frFj0=OqFfea0dXdd9vqai=hGuQ8kuc9pgc9s8qqaq=dirpe0xb9q8qiLsFr0=vr0=vr0dc8meaabaqaciaacaGaaeqabaWaaeGaeaaakeaaimaacaWFqbWaaSbaaSqaaiabdkgaIjabdwgaLjabdohaZjabdsha0bqabaaaaa@3F17@, we also add the appropriate suffix. So, *GenClust-Random-last *takes as input a random partition and returns the last partition produced during its execution. We also used an implementation of *Cast *that was made available to us by Ka Yee Yeung and that is well suited for the FOM methodology. Finally, we have used the version of *Click *available with the *Expander *software system [31].

#### Validation criteria

The adjusted Rand index measures the level of agreement between two partitions, not necessarily containing the same number of classes. Qualitatively, it takes value zero when the partitions are randomly correlated, value one when there is a perfect correlation, and value -1 when there is perfect anti-correlation. Those statements can be put on a more formal ground.

2-norm FOM, which is the internal measure used for our experiments, is a measure of the predictive power of a clustering algorithm. It should display the following properties. For a given clustering algorithm, it must have a low value in correspondence with the number of clusters that are really present in the data. Moreover, when comparing clustering algorithms for a given number of clusters *k*, the lower the value of 2-norm FOM for a given algorithm, the better its predictive power. Experiments by Ka Yee Yeung et al. show that the FOM family and its associated validation methodology satisfy those properties with a good degree of accuracy. Indeed, Ka Yee Yeung et al. give experimental evidence of some degree of anti-correlation between FOM and adjusted Rand index, in particular when the number of clusters is small. Since it is a rather novel measure, we provide a formal definition.

For a given data set, let *R *denote the raw data matrix, e.g., the data matrix without standardization for our data sets. Assume that *R *has dimension *nxm*, i.e., each row corresponds to a gene and each column corresponds to an experimental condition. Assume that a clustering algorithm is given the raw matrix *R *with column *e *excluded. Assume also that, with that reduced data set, the algorithm produces *k *clusters *C*_0_, ..., *C*_*k*-1_. Let *R*(*g*, *e*) be the expression level of gene *g *and *m*_*i*_(*e*) be the average expression level of condition *e *for genes in cluster *C*_*i*_. The 2-norm FOM with respect to *k *clusters and condition *e *is defined as:

FOM(e,k)=1n∑i=0k−1∑x∈Ci(R(x,e)−mi(e))2     (4)
 MathType@MTEF@5@5@+=feaafiart1ev1aaatCvAUfKttLearuWrP9MDH5MBPbIqV92AaeXatLxBI9gBaebbnrfifHhDYfgasaacH8akY=wiFfYdH8Gipec8Eeeu0xXdbba9frFj0=OqFfea0dXdd9vqai=hGuQ8kuc9pgc9s8qqaq=dirpe0xb9q8qiLsFr0=vr0=vr0dc8meaabaqaciGacaGaaeqabaqabeGadaaakeaacqqGgbGrcqqGpbWtcqqGnbqtcqGGOaakcqWGLbqzcqGGSaalcqWGRbWAcqGGPaqkcqGH9aqpdaGcaaqaamaalaaabaGaeGymaedabaGaemOBa4gaamaaqahabaWaaabuaeaacqGGOaakcqWGsbGucqGGOaakcqWG4baEcqGGSaalcqWGLbqzcqGGPaqkcqGHsislcqWGTbqBdaWgaaWcbaGaemyAaKgabeaakiabcIcaOiabdwgaLjabcMcaPiabcMcaPmaaCaaaleqabaGaeGOmaidaaaqaaiabdIha4jabgIGiolabdoeadnaaBaaameaacqWGPbqAaeqaaaWcbeqdcqGHris5aaWcbaGaemyAaKMaeyypa0JaeGimaadabaGaem4AaSMaeyOeI0IaeGymaedaniabggHiLdaaleqaaOGaaCzcaiaaxMaacqGGOaakcqaI0aancqGGPaqkaaa@5D8D@

Notice that FOM(*e*, *k*) is essentially a root mean square deviation. The aggregate 2-norm FOM for *k *clusters is then:

FOM(k)=∑e=1mFOM(e,k)     (5)
 MathType@MTEF@5@5@+=feaafiart1ev1aaatCvAUfKttLearuWrP9MDH5MBPbIqV92AaeXatLxBI9gBaebbnrfifHhDYfgasaacH8akY=wiFfYdH8Gipec8Eeeu0xXdbba9frFj0=OqFfea0dXdd9vqai=hGuQ8kuc9pgc9s8qqaq=dirpe0xb9q8qiLsFr0=vr0=vr0dc8meaabaqaciGacaGaaeqabaqabeGadaaakeaacqqGgbGrcqqGpbWtcqqGnbqtcqGGOaakcqWGRbWAcqGGPaqkcqGH9aqpdaaeWbqaaiabbAeagjabb+eapjabb2eanjabcIcaOiabbwgaLjabcYcaSiabbUgaRjabcMcaPaWcbaGaemyzauMaeyypa0JaeGymaedabaGaemyBa0ganiabggHiLdGccaWLjaGaaCzcaiabcIcaOiabiwda1iabcMcaPaaa@479D@

A few remarks are in order. Both formulae (4) and (5) can be used to measure the predictive power of an algorithm. The first gives us more flexibility, since we can pick any condition, while the second gives us a total estimate over all conditions. Following the literature, we use (5) in our experiments. Moreover, since the experimental studies conducted by Ka Yee Yeung et al. show that FOM(*k*) behaves as a decreasing function of *k*, an adjustment factor has been introduced to properly compare clustering solutions with different numbers of clusters. A theoretical analysis by Ka Yee Yeung et al. provides the following adjustment factor:

n−kn.     (6)
 MathType@MTEF@5@5@+=feaafiart1ev1aaatCvAUfKttLearuWrP9MDH5MBPbIqV92AaeXatLxBI9gBaebbnrfifHhDYfgasaacH8akY=wiFfYdH8Gipec8Eeeu0xXdbba9frFj0=OqFfea0dXdd9vqai=hGuQ8kuc9pgc9s8qqaq=dirpe0xb9q8qiLsFr0=vr0=vr0dc8meaabaqaciGacaGaaeqabaqabeGadaaakeaadaGcaaqaamaalaaabaGaemOBa4MaeyOeI0Iaem4AaSgabaGaemOBa4gaaaWcbeaakiabc6caUiaaxMaacaWLjaGaeiikaGIaeGOnayJaeiykaKcaaa@36CD@

When (6) divides (4), we refer to (4) and (5) as *adjusted *FOMs. We use the adjusted aggregate FOM for our experiments and, for brevity, we refer to it simply as FOM.

#### Experimental setup

All of the experiments were performed on a PC with 1G of main memory and a 3.2 GHZ AMD Athlon 64 processor. For the randomized algorithms, i.e., *Cast, GenClust-Random, K-means-Random*, we executed five runs to measure the variability of the validation measures with respect to the various solutions found by the algorithms. We find that only *K-means-Random *and *GenClust-Random-best *display a non-negligible variation from run to run, but for the adjusted Rand index only. For those algorithms and particular index, we report the minimum and the maximum value obtained in each run, while we give the results of a single run in all other cases.

### Experiments

We now analyze the performance of *GenClust*, with respect to the choice of the initial partition, the two partitions it can give in output, and the performance of the other algorithms.

#### Convergence to a local optimum of internal variance

**Figure 1:**
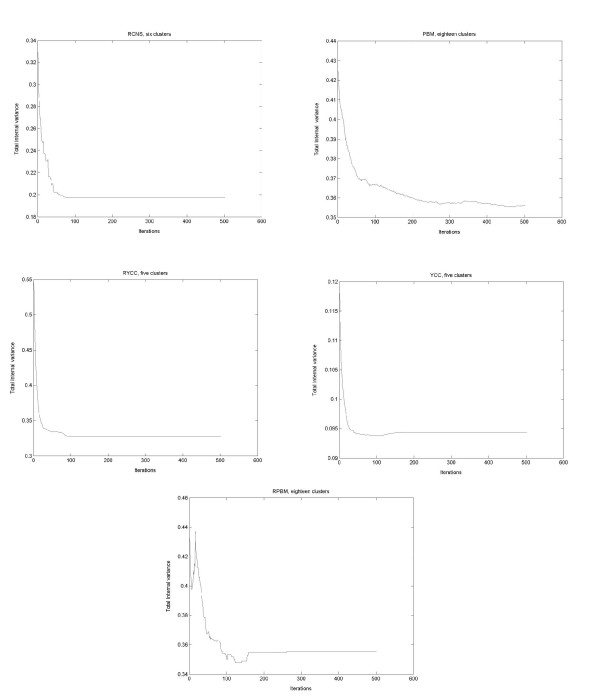
**Convergence of GenClust**. Experimental convergence of *GenClust *on each of the five data sets. The *x*-coordinate gives the number of iterations and the *y*-coordinate the value of the total internal variance (2). For each data set, the experiment was performed by asking the algorithm to return a clustering solution with a number of clusters equal to the number of classes in the true solution, for each data set.

For each of the chosen data sets, we have run *GenClust-Random-last *for 500 iterations; i.e., it has produced 500 partitions. The value of *k *has been set equal to the classes in the true solution for each data set. The results are reported in Figure 1. As is evident, such a convergence indeed takes place with a good degree of accuracy. It is also worth noting that for **RCNS, YCC **and **RYCC**, the convergence is rather fast, i.e., 100 iterations. For the remaining two data sets, it is somewhat slower and, for one of them, less pronounced. The same conclusions apply to *GenClust-Random-best.*

#### GenClust and the best and last partition

**Figure 2:**
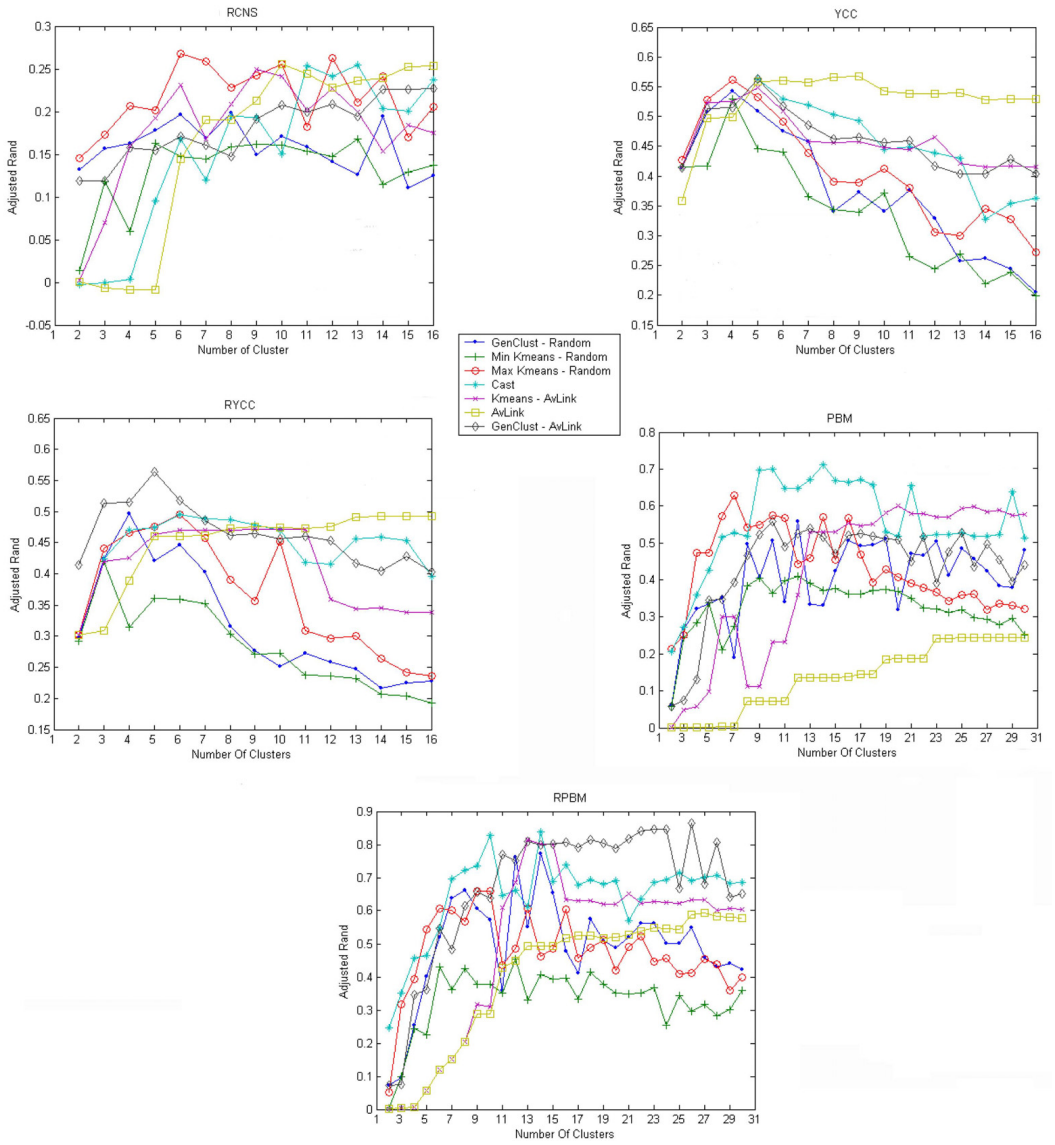
**Adjusted Rand Index**. Experiments for adjusted Rand index. For each data set and each algorithm, the index is displayed as a function of the number of clusters.

The discussion here refers to the data available at [30] (Figures 1 and 2), summarizing the experiments we conducted for *GenClust-Random-best *and *GenClust-AvLink-best*. This latter algorithm is really indistinguishable from *AvLink*. Indeed, it is not surprising that *GenClust-AvLink-best *retains the main characteristics of the initial partition given by *AvLink*, which, in our experiments, often provides an initial partition to *GenClust-AvLink-best *with the best variance. This fact seems to indicate that the partition corresponding to the best variance should not be required as output to *GenClust *if the initial partition is given by another clustering algorithm. *GenClust-Random-best *seems to be related to *K-means-Random*. Indeed, the relation is quite strong for FOM. As for the adjusted Rand index, the minimum values of the two algorithms are in many circumstances quite close. Such a relation is less pronounced for the maximum values, where sometimes one of the two algorithms dominates the other. There is, however, one important difference between the two algorithms: *GenClust-Random-best *is much faster than *K-means-Random*, e.g., four times faster on the **PBM **data set. The relation between the two algorithms seems to have the following justification. Starting from a random partition, *K-means-Random *tries to minimize the internal variance and, in practice, it aims at a good local optimum. *GenClust-Random-best *performs pretty much the same task by keeping track of the partition corresponding to the best variance seen during its execution. Based on those considerations, from now on we discuss only *GenClust-Random-last *and *GenClust-AvLink-last *and, for brevity, drop the suffix *last.*

#### A synopsis of GenClust performance for external and internal criteria

The values of interest are the adjusted Rand index and FOM. They have been computed requiring all algorithms, except *Click*, to produce a number of clusters equal to the classes of the *true solution *in each data set. The results are reported in Tables 1, 2, 3, 4, 5. Table 6 refers to *Click*, used in an unsupervised fashion, and for the adjusted Rand index. Indeed, *Click *does not lend itself to adaptation with the FOM methodology. Data has been given to *Click*, which has returned a partition. Since *Click *leaves elements unclustered, we have grouped all of those singletons together in one class in order to compute the adjusted Rand index. The number of classes in Table 6 accounts for that unification.

**Table 1:** RCNS Data Set. Performance of the algorithms at the number of classes (six) of the *true solution *for RCNS Rat data set.

*Method*	*AdjustedRand*	*FOM*
*GenClust random*	0.168	3.89
*Min kmeans-random*	0.144	3.81
*Max kmeans-random*	0.258	3.81
*Cast*	0.12	3.98
*Kmeans-Avlink*	0.167	3.71
*Avlink*	0.19	4.05
*GenClust-Avlink*	0.161	4.07

**Table 2:** YCC. Performance of the algorithms at the number of classes (five) of the *true solution *for YCC data set.

*Method*	*AdjustedRand*	*FOM*
*GenClust random*	0.47	57.05
*Min kmeans-random*	0.44	57.05
*Max kmeans-random*	0.49	57.05
*Cast*	0.529	56.66
*Kmeans-Avlink*	0.508	57.36
*Avlink*	0.559	58.78
*GenClust-Avlink*	0.518	57.21

**Table 3:** RYCC. Performance of the algorithms at the number of classes (five) of the *true solution *for the RYCC data set.

*Method*	*AdjustedRand*	*FOM*
*GenClust random*	0.446	10.60
*Min kmeans-random*	0.359	10.69
*Max kmeans-random*	0.49	10.69
*Cast*	0.49	10.84
*Kmeans-Avlink*	0.469	10.73
*Avlink*	0.46	11.50
*GenClust-Avlink*	0.518	10.804

**Table 4:** PBM. Performance of the algorithms at the number of classes (eighteen) of the *true solution *for the PBM data set.

*Method*	*AdjustedRand*
*GenClust random*	0.51
*Min kmeans-random*	0.37
*Max kmeans-random*	0.429
*Cast*	0.528
*Kmeans-Avlink*	0.58
*Avlink*	0.18
*GenClust-Avlink*	0.51

**Table 5:** RPBM. Performance of the algorithms at the number of classes (eighteen) of the *true solution *for the RPBM data set.

*Method*	*AdjustedRand*	*FOM*
*GenClust random*	0.509	57.49
*Min kmeans-random*	0.378	55.73
*Max kmeans-random*	0.51	55.73
*Cast*	0.679	50.21
*Kmeans-Avlink*	0.618	59.49
*Avlink*	0.517	62.27
*GenClust-Avlink*	0.80	59.33

**Table 6:** Adjusted Rand Index for Click. Performance of Click on the various data sets. The results in the clusters column give the number of clusters returned by Click, in addition to one class consisting of all the unclustered elements.

*Dataset*	*Clusters*	*AdjustedRand*
**RCNS**	3 + 1	0.183
**PBM**	18 + 1	0.767
**RPBM**	6 + 1	0.658
**YCC**	7 + 1	0.510
**RYCC**	6 + 1	0.479

The first striking conclusion is that no algorithm is markedly superior to the others on all indexes and all data sets. Indeed, in many cases the observed differences between the worst and best performing algorithm may be statistically insignificant and they could be considered equivalent. However, there are cases in which an algorithm may be better than others and therefore worthwhile.

Based on the synopsis, it appears that *GenClust-AvLink *is to be preferred to *GenClust-Random*. Moreover, *GenClust-AvLink *seems to take better advantage of the output of *Average Link *than *K-means*. It also appears that *GenClust-AvLink *is competitive, both in comparison with classic algorithms, i.e., *Average Link *and *K-means*, and more recent state-of-the-art ones, such as *Cast *and *Click*. The following present a detailed description of our experiments.

#### External criteria

This discussion refers to Figure 2. We recall from the literature that a good algorithm must display a good value of the Adjusted Rand Index for clustering solutions that have a number of clusters close to the classes of the *true solution*, for any given data set.

With that criterion in mind, we see that, with the exception of the **RCNS **data set, *GenClust *is better with an initial partition provided by *Average Link*, in particular around the number of clusters in the *true solution *of each of the corresponding data sets.

Moreover, on the **YCC, RYCC **and **RPBM **data sets, *GenClust *seems to take better advantage than *K-means *of the initial knowledge of the partition produced by *Average Link*.

When compared with all of the methods, *GenClust-AvLink *has a performance at least as good, and sometimes better, on three of the data sets, i.e., **YCC, RYCC **and **RPBM, **around the number of classes in the true solution of each data set.

#### Internal criteria

**Figure 3:**
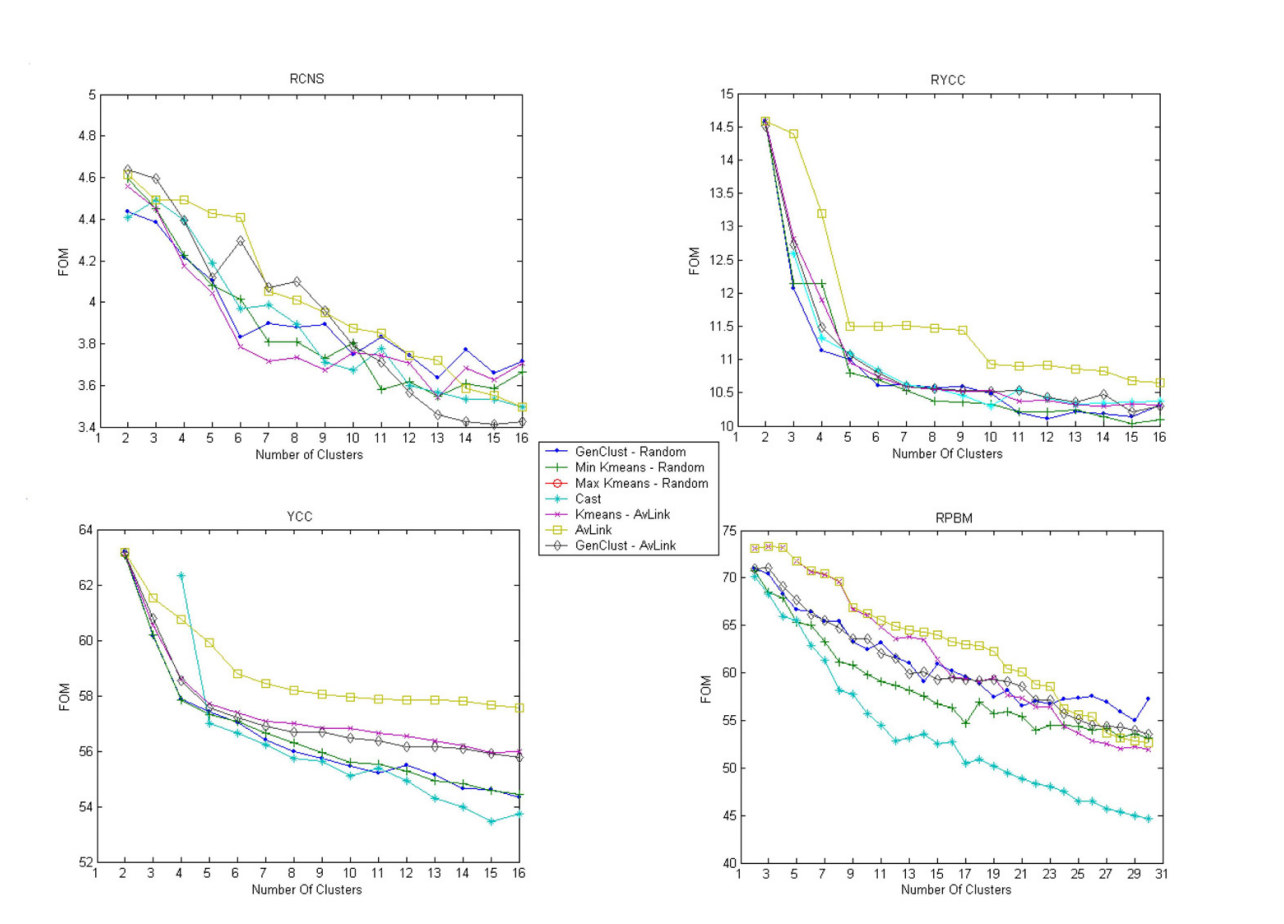
**FOM**. Experiments for FOM. The index is displayed as a function of the number of clusters.

This discussion refers to Figure 3. We recall from the literature [11,12] that the FOM methodology captures the intrinsic structure in the data by exhibiting a very characteristic steep decline as the number of clusters grows and approaches the number of clusters in the *true solution*. For our data sets, we find that all partitional algorithms exhibit excellent predictive power on the **RCNS, YCC **and **RYCC. **In particular, the curve of each algorithm indicates that the number of clusters really present in the data is close or at exactly the number of classes in the true solution of each data set. Moreover, when the *GenClust *curves are excluded from the FOM diagrams, the results are essentially analogous to the ones reported in [11,12] for the same algorithms on essentially the same data sets. Since in [11,12] it is concluded that *K-means *and *Cast *have excellent predictive power, we can draw the same conclusion for both versions of *GenClust*. As for the **RPBM, **we see that all algorithms do not exhibit any noticeable decline as the number of clusters grows. This may be a limitation of the FOM methodology, which displays some anti-correlation with the adjusted Rand index only for data sets with a small number of clusters in the *true solution*, as shown by Ka Yee Yeung et al. In fact, the internal validation of **PBM **and **RPBM **attempted here may indicate both a computational and sensitivity limitation of the FOM methodology; i.e., a data set with relatively large numbers of conditions and genes and a large number of clusters. Indeed, the external validation measure on both data sets shows that *GenClust *picks a substantial part of the true solution at a number of clusters reasonably close to 18. In general, any algorithm such as *GenClust *and *Kmeans*, will be limited by the power of the validation methodology associated to it. Valid alternatives to FOM are given in [8,9]. In particular, Monti et al. provide a good presentation of those alternatives. Unfortunately, the data driven measures may display the same computational limitations displayed by FOM. Principal component analysis, a widely used data dimensionality reduction technique for clustering, may be of great help to reduce the computational demand of data driven validation measures. Unfortunately, its application to gene expression data is not entirely straightforward. This point is investigated experimentally in Ka Yee Yeung doctoral dissertation, where different strategies are proposed and compared. In those circumstances, it is also advisable to filter the data set, for instance with the *GeneCluster *software package [20], leaving out genes that do not display any significant changes. That may result in a substantial reduction of the data set, as shown Ka Yee Yeung et al. in the analysis of the Barrett Esophagus data set.

## Conclusion

We have presented a very simple genetic algorithm for clustering of gene expression data, i.e., *GenClust*, and we have evaluated its performance on real data sets and in comparison with other either classic or more state-of-the-art algorithms, with use of both external and internal validation criteria. The study shows that none of the chosen algorithms is clearly superior to the others in terms of ability to identify classes of truly functionally related genes in the given data sets. However, *GenClust *seems to be competitive with all of the implemented algorithms and well suited for use in conjunction with the data driven internal validation measures, as the experiments with FOM indicate.

## Availability and requirements

- **Project Name: ***GenClust*

- **Project Home Page: **

- **Operating Systems: **Windows XP, Mac OSX, Linux Operating Systems (see details at [30]).

- **Programming Languages: **Standard ANSI C. Compilation tested on Microsoft Visual C++ 6, Pelles C for Windows-version 3.00.4, and various gcc versions (see [30]).

- **Other Requirements: **None

- **License: **GNU GPL

- **Any restriction to use by non-academics: **reference to paper

## Abbreviations

FOM: Figure of Merit

**PBM: **Pheripheral Blood Monocytes

**RPBM: **Reduced Pheripheral Blood Monocytes

**RCNS: **Central Nervous System Rat

**RYCC: **Reduced Yeast Cell Cycle

**YCC: **Yeast Cell Cycle

## Authors' contributions

All authors participated in the design of the evaluation of *GenClust*. The initial design and engineering of the algorithm is due to G. Lo Bosco and V. Di Gesú. D. Scaturro and A. Raimondi realized part of the software needed for the comparative analysis of *GenClust*. R. Giancarlo coordinated the research and wrote the report. All authors have read and approved the manuscript.
